# The significance of pre-therapeutic F-18-FDG PET–CT in lymphoma-associated hemophagocytic lymphohistiocytosis when pathological evidence is unavailable

**DOI:** 10.1007/s00432-015-2094-z

**Published:** 2015-12-15

**Authors:** Jujuan Wang, Dongjiao Wang, Qingbo Zhang, Limin Duan, Tian Tian, Xiaoyan Zhang, Jianyong Li, Hongxia Qiu

**Affiliations:** 1 Department of Hematology, The First Affiliated Hospital of Nanjing Medical University, Jiangsu Province Hospital, 300 Guangzhou Road, Nanjing, 210029 China; 2 Department of PET-CT Centre, The First Affiliated Hospital of Nanjing Medical University, Jiangsu Province Hospital, 300 Guangzhou Road, Nanjing, 210029 China; 3 Department of Geriatrics, The First Affiliated Hospital of Nanjing Medical University, Jiangsu Province Hospital, 300 Guangzhou Road, Nanjing, 210029 China

**Keywords:** Lymphoma-associated hemophagocytic lymphohistiocytosis (LAHLH), PET–CT, Differential diagnosis, Chemotherapy, Prognosis

## Abstract

**Purpose:**

The significance of positron emission tomography/computed tomography (PET–CT) in identifying patients with lymphoma-associated hemophagocytic lymphohistiocytosis (LAHLH) when pathological evidence is unavailable remains uncertain.

**Methods:**

In this retrospective study, 44 HLH patients who underwent PET–CT before clinical treatment were enrolled, and 18 of them were highly suspected as LAHLH by PET–CT. We compared the PET–CT parameters between confirmed LAHLH and non-LAHLH patients. The efficacy of initial therapies for highly suspected LAHLH patients was analyzed as well.

**Results:**

We found that the SUV_Sp_, SUV_BM_, SUV_LN_, SUV_max_, SUV_LN/Li_, and SUV_max/Li_ in LAHLH group were significantly higher than those in non-LAHLH group (*p* = 0.003, *p* = 0.034, *p* = 0.003, *p* < 0.001, *p* = 0.039, and *p* = 0.035, respectively). HLH patients with an SUV_max_ value >5.5, an SUV_LN_ value >3.3, and an SUV_Sp_ value >4.8 were more likely to be LAHLH (*p* < 0.001, *p* = 0.003, and *p* = 0.003, respectively). And the incidence of multiple lymphadenopathy with increased FDG uptake or the incidence of multiple bone lesions in LAHLH patients was significantly higher than those in non-LAHLH group (92.9 vs. 35.7 %, *p* = 0.004; 42.9 vs. 0 %, *p* = 0.016, respectively). Furthermore, by comparing the efficacy of initial therapies for highly suspected LAHLH patients (*n* = 18), we indicated that the CR rate was significantly higher in lymphoma-chemotherapy group than in immunosuppressive therapy group (90 and 25 %, respectively; *p* = 0.013). OS analysis revealed that highly suspected LAHLH patients treated with lymphoma-chemotherapy had better prognosis (264 days) than those treated with immunosuppressive therapy (15 days) (*p* < 0.0001).

**Conclusions:**

When pathological evidence is absent, PET–CT may play an important role in identifying HLH patients underlying lymphoma. Once highly suspected as LAHLH by PET–CT, lymphoma-chemotherapies that directly treat the underling lymphoma may have a relatively favorable effect and better clinical outcomes than immunosuppressive therapy.

## Introduction

Hemophagocytic lymphohistiocytosis (HLH) is a life-threatening syndrome of hyper-inflammation, caused by uncontrolled activation and proliferation of lymphocytes and antigen-presenting cells (macrophages, histiocytes) (Filipovich 2009). The disrupted critical regulatory pathways, which are responsible for the natural termination of immune responses, result in burst release of cytokines. The flooding of cytokines, such as IL-6, IL-12, TNF-α, and IFN-γ (Emmenegger et al. 2005; Szyper-Kravitz 2009), leads to the typical clinical manifestations, such as hepatosplenomegaly, persistent high fever, cytopenia, dysfunction of liver and hepatitis. In addition, the rapid progression and extremely high mortality rate of HLH despite proper management (Kaito et al. 1997) frustrate both patients and physicians.

Based on the presence or absence of hereditary disorder, HLH can be classified into primary HLH and secondary HLH. Primary HLH, known as familial HLH (FHL), is a disease caused by mutations in the genes with a median survival of less than 2 months after diagnosis if not treated (Henter et al. 2007). The first onset of FHL is at infancy or at early childhood (Henter et al. 2007). Secondary HLH (sHLH) may develop as a result of strong immune activation in association with severe infections, malignancies, or autoimmune diseases. Accordingly, it is, respectively, classified as infection-associated HLH (IAHLH), malignancy-associated HLH (MAHLH) (Janka et al. 1998), and rheumatic autoimmune diseases-associated HLH (RAHLH). In addition, there are some idiopathic HLH patients, from whom confirmed etiological factors are unavailable (Kim et al. 2013). Further studies about identifying the causative agents and finding efficacious regimens to these specific sHLH patients are seldom reported.

F-18 fluoro-2-deoxyglucose positron emission tomography/computed tomography (F-18 FDG PET–CT) is a functional imaging technique. It can not only correctly describe the morphologic and metabolic states of the lesions, but also check the infiltrated lymph nodes, spleen, bone marrow, and other extranodal organs, which are usually neglected by conventional imaging examinations (de Jong et al. 2009). Therefore, it plays an important role in the diagnosis, staging, post-therapeutic follow-up, and prognosis of many malignancies. In the case of lymphoma, PET identified >97 % of disease sites of Hodgkin lymphoma (HL) and aggressive and highly aggressive non-Hodgkin lymphoma (Henter et al. 2007). It also localizes suitable sites of biopsy that is accurate for baseline staging in the absence of palpable lymphadenopathy, and yields important prognostic information for determining the most appropriate initial treatment (Cronin et al. 2010).

So far, little research has described the characteristic findings of F-18 FDG PET–CT in patients with HLH. Our previous study reported that the maximum standardized uptake value (SUV_max_) of patients in MAHLH group was significantly higher than of patients in IAHLH group or RAHLH group (Zhang et al. 2012). Therefore, we hypothesized that, for sHLH patients with out known etiology, F18-FDG PET–CT might provide beneficial data in distinguishing lymphoma-associated HLH (LAHLH) patients and in determining a better initial therapy.

To research these issues, the aims of the present study were to:

1. Investigate the characteristics of F-18 FDG PET–CT image in patients with sHLH. Find out the differences of F-18 FDG PET–CT characteristics between LAHLH and non-lymphoma-associated HLH (non-LAHLH).
2. Discuss the diagnostic performance of PET–CT for the detection of underlying lymphoma, especially when pathological evidences were unavailable.
3. Compare the efficacy of different regimens for LAHLH without pathological basis. Explore the significance of PET–CT in choosing better treatment strategy for idiopathic HLH patients.

## Patients and methods

### Patients

There were total of 44 adult patients newly diagnosed with HLH who had undergone F-18 FDG PET–CT scanning before clinical treatment between February 2011 and December 2014. They were enrolled in this retrospective analysis. HLH was diagnosed according to the diagnostic criteria of HLH-2004 protocol (Group 2004). In order to ascertain the diagnosis of HLH and find out possible underlying diseases, a series of laboratory tests and equipment inspections were selectively and systematically performed, including blood, bone marrow, or sputum culture: PCR tests of virus DNA loads (such as Epstein–Barr virus, cytomegalovirus, hepatitis virus); screening of tumor markers and autoimmune indicators; CT scans; bone marrow aspiration and biopsy; and lymph node biopsy in some cases. Fourteen of these patients were identified as lymphoma-associated HLH (LAHLH) (among them, nine had a pathological basis of lymphoma at first visit and five lacked evidence of lymphoma in the early stage of the disease and confirmed diagnosis after chemotherapy started), 11 patients as IAHLH, three patients as RAHLH. The three RAHLH patients met authoritative diagnostic criteria after combining clinical symptoms and signs with laboratory tests and imaging findings. No etiology factors were found in the other 16 patients. The distribution of etiologies of the 44 patients with HLH is presented in Table 1.

**Table 1:** Distribution of etiologies in 44 patients with HLH

Etiologies	Number
Unexplained HLH	16 (36.4 %)
Highly suspected lymphoma by PET–CT	13
Others	3
Lymphoma-associated HLH	14 (31.8 %)
DLBCL	4
T cell lymphoma	4^a^
Peripheral T cell lymphoma	2^b^
B cell lymphoma	2^c^
Hodgkin’s lymphoma	1
NK/T cell lymphoma	1
Infection-associated HLH	11 (25.0 %)
EBV	5
CMV	1
Herpes virus	1
Pulmonary infection	3
Staphylococcus sepsis	1
Autoimmune disorder-associated HLH	3 (6.8 %)
Adult Still’s disease	1
Systemic lupus erythematosus	1
Sjogren’s syndrome	1

^a^Three cases of T cell lymphoma, ^b^ one case of peripheral T cell lymphoma, and ^c^ one case of B cell lymphoma who lacked pathological evidence initially were highly suspected as lymphoma by PET–CT (*n* = 5). They were confirmed as lymphoma according to their pathological evidence which were got after treatment or in the end stage of the disease

For each patient, we collected the following information: age, sex, presence of fever and splenomegaly, routine blood count tests, blood biochemical tests (including triglyceride, aspartate aminotransferase, alanine aminotransferase, and lactate dehydrogenase), fibrinogen, serum ferritin, serum soluble interleukin-2 receptor (sIL-2R, sCD25), C-reactive protein (CRP), and lymphocyte subsets tests including proportion of CD3+, CD3+/CD4+, CD3+/CD8+, CD3-CD16+CD56+. Bone marrow aspiration and biopsy were done at the time of initial diagnosis. Type of initial therapy and outcome of treatment were also reviewed.

### Image acquisition

The F-18 FDG PET–CT images were acquired from the skull base to the upper thigh with a special-purpose PET–CT scanner (Discovery ST, GE Healthcare). Before the examination was done, patients were asked to be fasting for at least 6 h to control blood glucose level lower than 7.0 mmol/L. All patients received an F-18 FDG dose of 3.7–5.55 MBq/kg by intravenous injection. In a quiet state, the distribution of this tracer in the human body needed a period of time, and the mean time from F-18 FDG injection to imaging was 60 min. CT data were used for attenuation correction, and images were reconstructed by using a conventional iterative algorithm. Tomographic slice thickness was designed as 5 mm. Ultimately, fusion images of PET and CT on cross-sectional plane, sagittal plane, and coronal plane were obtained. The standardized uptake value (SUV) is the measured activity at the region of interest, which is normalized for body weight/surface area and injected dose.

### Image analysis

PET–CT images were reviewed by experienced nuclear medicine physicians on a dedicated workplace. To measure the maximum SUV in the spleen and liver, round (or elliptical) regions of interest (ROIs) were drawn on the center of the spleen or right lobe of the liver, respectively. Also round ROIs were drawn over the thoracic (T10–12) and lumbar (L2–4) vertebral bodies and enlarged lymph nodes to measure bone marrow and lymph nodes activity. Then, the maximum SUV of the bone marrow, spleen, lymph nodes, and liver was described in our study as SUV_BM_, SUV_Sp_, SUV_LN_, and SUV_Li_, respectively. Excluded the sites of which increased SUV value were considered as physiological uptake, the highest whole body SUV value was set as SUV_max_. SUV_BM/Li_ was calculated by dividing SUV_BM_ by SUV_Li_. SUV_Sp/Li_ and SUV_max/Li_ were calculated as the same.

Other information of PET–CT findings which could not be described by SUV levels was collected. The presence of splenomegaly, hepatomegaly, pulmonary inflammatory lesions, liver lesions, and effusions; sites and numbers of lymphadenopathy and bone lesions were enrolled. The presence of lymphadenopathy in three or more lymph node regions was defined as multiple lymphadenopathy. Bone lesions were referred to bone sites where SUV level increased. Degenerative changes such as hyperosteogeny were excluded. Similarly, the presence of increased FDG uptake in three or more bone regions was defined as multiple bone lesions. Pulmonary inflammatory lesions were referred to pneumonia, atelectasis or bronchiectasis. And liver cyst and fatty liver were included in liver lesions.

### Treatment group

A total of 13 HLH patients who had no clear etiology factor were highly suspected as lymphoma by PET–CT detection. And another five confirmed lymphoma-associated patients who lacked pathological evidence in the early stage of the disease were highly suspected as lymphoma by PET–CT, as well. In all, 18 patients were highly suspected as LAHLH by initial PET–CT. For further research of the initial therapeutic regimen of patients who were highly suspected as LAHLH by PET–CT without clear pathological evidences, they were grouped according to their initial therapy. Suspected LAHLH patients treated with chemotherapy (*n* = 13), which usually applied to lymphoma, were enrolled in lymphoma-chemotherapy group. Other suspected LAHLH patients treated with immunosuppressive therapy (*n* = 5) which was mainly focused on suppressing excessive immune response and cytokine storm were enrolled in immunosuppressive therapy group.

### Treatment regimen

#### Initial therapy

In lymphoma-chemotherapy group, eight patients received EPOCH chemotherapy (Gutierrez et al. 2000) or dose-adjusted EPOCH (DA-EPOCH) chemotherapy (Wilson et al. 2008), four patients adopted CHOP chemotherapy (Pfreundschuh et al. 2008), and one patient was given hyper-CVAD-A chemotherapy (Thomas et al. 2004). Extensional schemes are not described in detail here.

Protocols that were aimed directly at super cytokine storm and hyper-inflammation were used in five patients of immunosuppressive therapy group. They were given high-dose corticosteroid combined with intravenous immunoglobulin (IVIG) pulse therapy, which was consisted of Dex 10 mg/(m^2^ day) i.v. daily and immunoglobulin 0.4 g/(kg day) for 5 days.

#### Supportive therapy

According to bacterial or fungal culture results, appropriate or even experienced broad-spectrum antibiotics and antimycotics were used. Antiviral drugs were included in the supportive therapy in infected patients. IVIG and intravenous human albumin were used when required. Liver protection and treatment to jaundice were performed. Blood component transfusion, including erythrocyte suspension, platelets, fresh-frozen plasma, and cryoprecipitate, was adopted. In some cases, granulocyte colony-stimulating factor (G-CSF), erythropoietin (EPO), and thrombopoietin (TPO) were given as needed. Certainly, proper supportive care was also essential.

### Treatment response evaluation

Clinical response (CR) was defined as explicit fulfillment of all criteria including: (1) no fever; (2) reduction in spleen size; (3) platelets >100 × 10^9^/L; (4) normal fibrinogen; (5) decreasing ferritin levels (by 25 %) (Group 2004). No response (NR) was considered if not all criteria were fulfilled. The evaluation of disease status was done 1–2 weeks after finishing the initial therapy.

### Statistical analysis

Statistical analyses were performed with commercial software package (SPSS, version 17.0, Chicago, IL, USA). Statistical significance was determined at a level of *p* < 0.05. PET–CT parameters of LAHLH group and non-LAHLH group were compared using Mann–Whitney *U* test. Occurrence rate of other PET–CT findings in LAHLH and non-LAHLH group was compared using Fisher’s exact test. To identify an optimal *cutoff* for each PET–CT parameter, receiver operating characteristic (ROC) analysis was performed. Correlations between PET parameters and laboratory parameters were assessed using the Spearman correlation test. The overall survival (OS) was calculated from the time of diagnosis to the time of death or last follow-up and was analyzed combined with survival curves using the Kaplan–Meier method. Differences between curves were tested using the log-rank test.

## Results

### PET–CT characteristics of sHLH and differences of PET–CT characteristics between LAHLH and non-LAHLH

We summarized the PET–CT characteristics of the 44 patients with sHLH who were admitted into our hospital and underwent the PET–CT examination. The male to female ratio was 1.25, and medium age was 42 years (29–60). All of the patients had at least three organs involved, including 37 cases (82.2 %) showing splenomegaly (among them 28 cases with increased SUV_Sp_), 35 cases (77.8 %) with bone lesions, 34 cases (75.6 %) lymphadenopathy (31 cases with increased SUV_LN_), 35 cases (77.8 %) inflammatory changes in the lung including pneumonia and/or atelectasis, 29 cases (64.4 %) serous effusions (in pleural cavity, peritoneal cavity, pelvic cavity, and/or pericardial cavity), 26 cases (57.8 %) pleura thickening and/or pleura adhesion, 17 cases (37.8 %) cholecystitis or cholelithiasis, 16 cases (35.6 %) sinusitis, 15 cases (33.3 %) heart lesions (mainly lower density of the heart chamber than the heart wall), 13 cases (28.9 %) brain tissue or cerebrovascular lesions, 10 cases (22.2 %) hepatomegaly (all accompanied by increased SUV_Li_, another three cases revealed no hepatomegaly but increased SUV_Li_), and eight cases (17.8 %) pericardium thickening. Other involved organs were kidney, mammary gland, muscles, oropharynx, and adnexa uteri.

To better distinguish LAHLH patients from HLH patients, patients were divided into three groups: LAHLH group (*n* = 14), non-LAHLH group (*n* = 14) (including IAHLH and RAHLH), and unexplained causes group (*n* = 16). We compared the PET–CT parameters between LAHLH and non-LAHLH patients, including SUV_Sp_, SUV_Li_, SUV_BM_, SUV_LN_, SUV_max_, SUV_Sp/Li_, SUV_BM/Li_, SUV_LN/Li_, and SUV_max/Li_. Nonparametric test showed that the levels of SUV_Sp_, SUV_BM_, SUV_LN_, SUV_max_, SUV_LN/Li_, and SUV_max/Li_ in LAHLH group were significantly higher than those in non-LAHLH group (*p* = 0.003, *p* = 0.034, *p* = 0.003, *p* < 0.001, *p* = 0.039, and *p* = 0.035, respectively). However, the levels of SUV_Li_, SUV_Sp/Li_, and SUV_BM/Li_ in two groups revealed no significant difference. The medians and interquartile ranges of different PET–CT parameters are given in Table 2.

**Table 2:** PET–CT parameters in LAHLH and non-LAHLH group

PET–CT parameter	Group		*p* value
	LAHLH (*n* = 14)	Non-LAHLH (*n* = 14)	
SUV_Sp_	5.7 (2.7–10.8)	2.0 (1.8–3.6)	0.003**
SUV_BM_	6.9 (2.2–10.0)	2.5 (2.0–3.7)	0.034*
SUV_Li_	2.1 (1.9–8.1)	2.0 (1.7–2.2)	0.079
SUV_LN_	10.6 (3.3–13.6)	2.3 (1.9–3.5)	0.003**
SUV_max_	12.8 (8.2–16.2)	4.3 (2.7–5.2)	0.000**
SUV_Sp/Li_	1.4 (1.0–2.7)	1.0 (0.8–1.6)	0.057
SUV_BM/Li_	1.5 (0.9–4.4)	1.2 (0.9–1.8)	0.581
SUV_LN/Li_	2.3 (1.1–6.5)	1.1 (0.9–1.6)	0.039*
SUV_max/Li_	4.3 (1.7–6.5)	2.3 (1.1–2.8)	0.035*

* Difference of PET–CT parameter between two groups is significant at *p* < 0.05

** Difference of PET–CT parameter between two groups is significant at *p* < 0.01

ROC curves demonstrated that a SUV_max_ of 5.5, a SUV_LN_ of 3.3, and a SUV_Sp_ of 4.8 were the optimal cutoffs to distinguish LAHLH patients from non-LAHLH patients. HLH patients with an absolute SUV_max_ value >5.5 were more likely to be LAHLH than those with a lower SUV_max_ value (*p* < 0.001, sensitivity = 92.9 % and specificity = 85.7 %, AUC = 0.923). Similarly, HLH patients with an absolute SUV_LN_ value >3.3 or an absolute SUV_sp_ value >4.8 were more likely to be LAHLH (*p* = 0.003, sensitivity = 78.6 % and specificity = 78.6 %, AUC = 0.834; *p* = 0.003, sensitivity = 71.4 % and specificity = 100 %, AUC = 0.832) (see Table 3).

**Table 3:** ROC analysis of PET–CT parameters in distinguishing LAHLH patients (*n* = 14) from non-LAHLH patients (*n* = 14)

	Cutoff	AUC	Sensibility (%)	Specificity (%)	*p* value
SUV_Sp_	4.8	0.832	71.4	100	0.003**
SUV_BM_	4.5	0.735	64.3	92.9	0.035*
SUL_Li_	6.4	0.694	28.6	100	0.081
SUV_LN_	3.3	0.834	78.6	78.6	0.003**
SUV_max_	5.5	0.923	92.9	85.7	0.000**
SUV_Sp/Li_	1.0	0.712	92.9	42.9	0.057
SUV_BM/Li_	1.7	0.561	50.0	78.6	0.581
SUV_LN/Li_	1.4	0.730	71.4	78.6	0.039*
SUV_max/Li_	2.8	0.735	64.3	78.6	0.035*

* Significance at *p* < 0.05

** Significance at *p* < 0.01

Table 4 shows the positive rate of other PET–CT findings in LAHLH and non-LAHLH group. Thirteen out of 14 (92.9 %) patients in LAHLH group and five out of 14 (35.7 %) patients in non-LAHLH group had multiple lymphadenopathy accompanied by increased FDG uptake. The incidence of multiple lymphadenopathy accompanied by increased FDG uptake in LAHLH patients was significantly higher than those in non-LAHLH group (*p* = 0.004). Six out of 14 (42.9 %) LAHLH patients had multiple bone lesions, and none of the non-LAHLH patients (0 %) had multiple bone lesions. The incidence of multiple bone lesions in LAHLH patients was significantly higher than those in non-LAHLH group (*p* = 0.016). No significant difference was found in the incidence of splenomegaly, hepatomegaly, pulmonary inflammatory lesions, liver lesions, and effusions between the two groups.

**Table 4:** Positive rate of other PET–CT findings in LAHLH and non-LAHLH group

PET–CT findings	Group		*p* value
	LAHLH (*n* = 14)	Non-LAHLH (*n* = 14)	
Splenomegaly	13/14 (92.9 %)	9/14 (64.3 %)	0.165
Hepatomegaly	2/14 (14.3 %)	2/14 (14.3 %)	1
Multiple lymphadenopathy	13/14 (92.9 %)	5/14 (35.7 %)	0.004**
Multiple bone lesions	6/14 (42.9 %)	0/14 (0 %)	0.016**
Pulmonary inflammatory lesions	10/14 (71.4 %)	12/14 (85.7 %)	0.648
Liver lesions (hepatic cyst and fatty liver)	6/14 (42.9 %)	4/14 (28.6 %)	0.695
Effusions	7/14 (50.0 %)	11/14 (78.5 %)	0.236

** Significance at *p* < 0.01

### Clinical characteristics, treatment outcome, and survival analysis

The cardinal features of the 18 highly suspected LAHLH patients were persistent high fever, pancytopenia, raised transaminases, hyperbilirubinemia, respiratory failure, and DIC tendency. They also had manifestations of lymphoma, such as night sweats, weight loss, enlarged lymphohematopoietic organs, and markedly elevated LDH and β2-microglobulin. Imaging examinations showed lymphoma-like lesions in PET–CT, as well. However, due to the absence of enlarged superficial nodes and rapid deterioration of vital signs, biopsies of deep lymph nodes or splenic resection were unavailable.

**Fig. 1:**
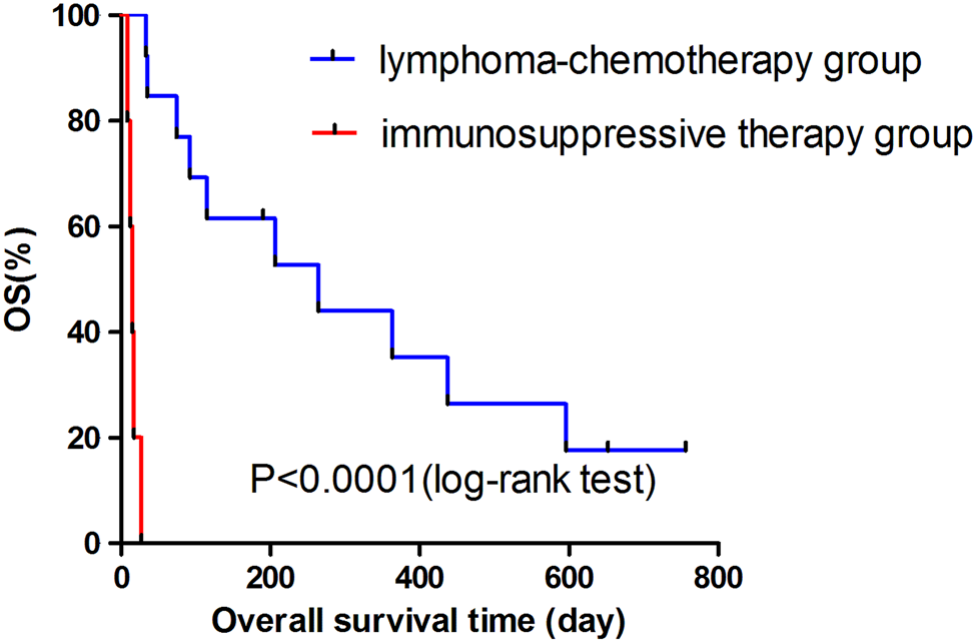
Kaplan–Meier survival of 18 highly suspected LAHLH patients treated with lymphoma-chemotherapy and immunosuppressive therapy. Kaplan–Meier analysis revealed a significant difference on OS between lymphoma-chemotherapy group and immunosuppressive therapy group by log-rank test (*p* < 0.0001)

The cutoff time was April 30, 2015. The median survival time of 18 patients who were highly suspected as LAHLH by initial PET–CT was 92 days (interquartile range, 27–436 days). Table 5 shows the response rate and median survival time of two groups. Ten out of 13 patients showed clinical response after initial lymphoma-chemotherapy; however, none showed clinical response in the immunosuppressive therapy group. The clinical response rate was significantly higher in lymphoma-chemotherapy group than in immunosuppressive therapy group (76.9 and 0 %, respectively; *p* = 0.007). The median survival time of lymphoma-chemotherapy group and immunosuppressive therapy group was 264 and 15 days, respectively. OS analysis revealed a significant difference on survival rate between the two groups by log-rank test (*p* < 0.0001) (Fig. 1). Therefore, for initial regimen of highly suspected LAHLH, lymphoma-chemotherapy has more curative efficacy than immunosuppressive therapy.

**Table 5:** Response rate of initial treatment and median survival time of 18 patients who were highly suspected as LAHLH by preliminary PET–CT

Group	Response		Median survival time (days), median (interquartile range)
	CR	NR	
Lymphoma-chemotherapy group	10/13 (76.9 %)	3/13 (23.1 %)	264 (92–596)
Immunosuppressive therapy group	0/5 (0 %)	5/5 (100 %)	15 (12–17)
*p* value	*p* = 0.007**		

Lymphoma-chemotherapy group refers to suspected LAHLH patients treated with lymphoma-chemotherapy; immunosuppressive therapy group refers to suspected LAHLH treated with high-dose corticosteroid and IVIG

** Significance at *p* < 0.01

**Fig. 2:**
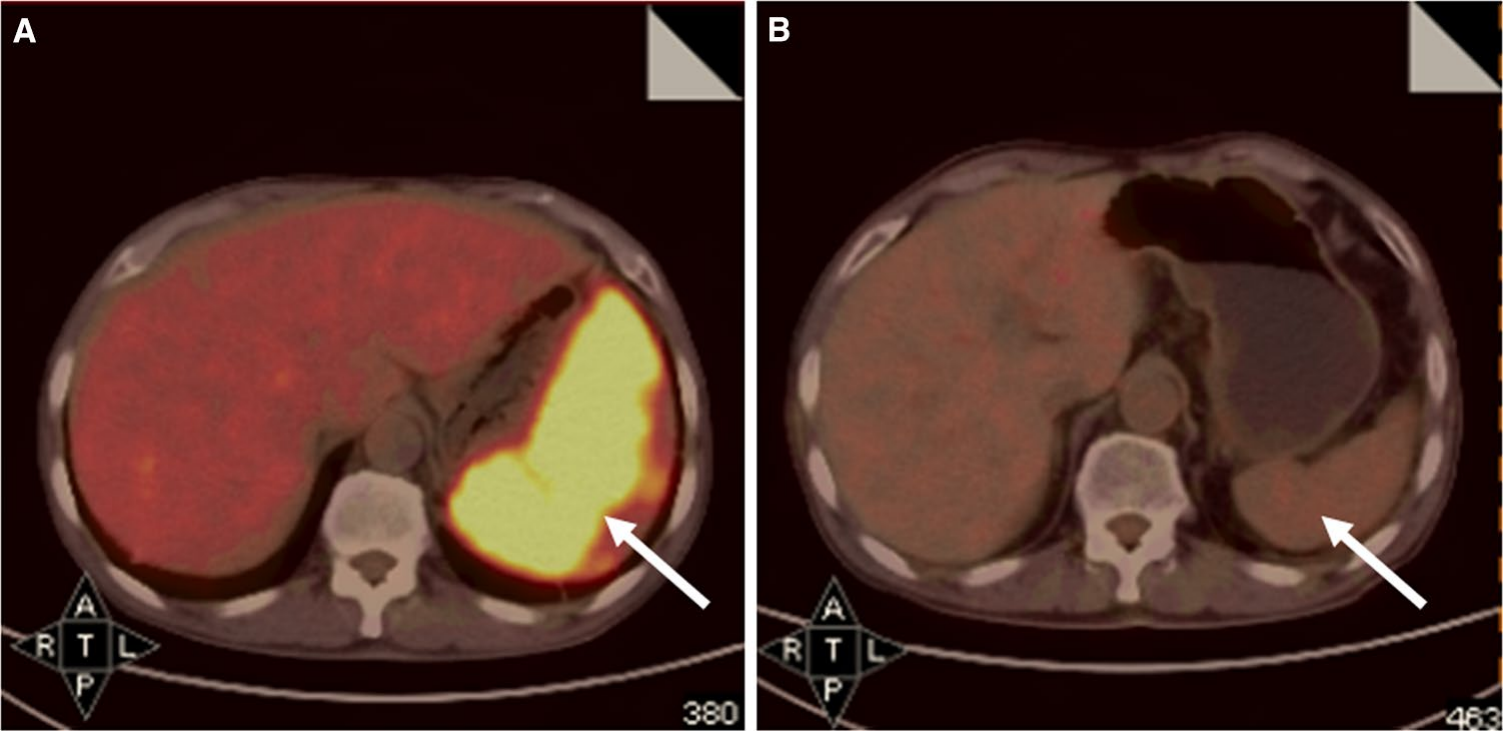
Comparison of PET–CT images of patient no. 2 who was highly suspected as lymphoma-associated HLH by PET–CT before lymphoma-chemotherapy and after treatment. **a** Initial PET–CT scan revealed FDG-avid splenomegaly with a SUV_Sp_ of 10.5 but no disease elsewhere (*arrow*). **b** Follow-up PET–CT scan was performed after receiving two cycles of EPOCH chemotherapy. It revealed significantly reduced spleen with no FDG-hypermetabolism (*arrow*). Until the date of cutoff time, the patient has survived for 652 days

**Fig. 3:**
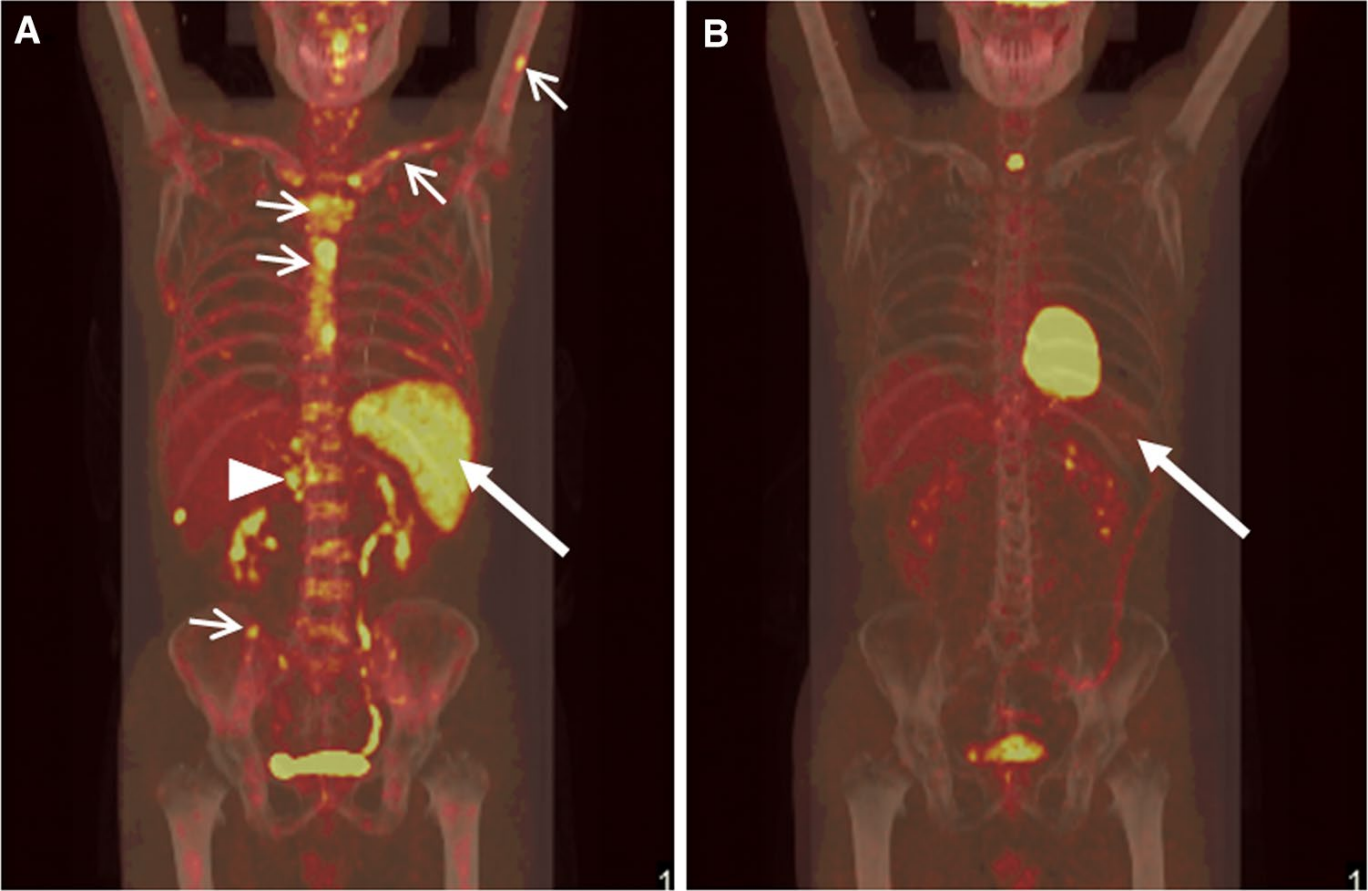
Comparison of PET–CT images of patient no. 9 before treatment and after lymphoma-chemotherapy. **a** Pre-therapeutic coronal PET–CT showed multiple FDG-avid lymph nodes around abdominal aorta (*triangle*), diffuse FDG uptake within enlarged spleen (*arrow*), and patchy FDG-avid lesions in sternum, clavicles, humerus, iliac bones, and multiple vertebral bodies (*arrowhead*). **b** After administered six cycles of DA-EPOCH chemotherapy, PET–CT revealed resolution of the metabolically active disease in multiple lymph nodes, spleen, and multiple bones, suggesting favorable efficacy of the previous chemotherapies. Until the date of cutoff time, the patient has survived for 264 days

Table 6 shows treatment courses and prognosis of highly suspected LAHLH patients receiving different initial treatment. Patients received CR and had a long survival time, except patient no. 8 who revealed no response to chemotherapy and died 1 month later, and patient no. 4 who got CR after initial chemotherapy but died of interruption of continuation therapy. Among these, post-therapeutic PET–CT was done on five patients (patient no. 2, 3, 5, 9, and 10) after completion of initial chemotherapy or several cycles of chemotherapy. Complete or major resolution of FDG-avid lesions was observed including spleen, liver, lymph nodes, and bone, suggesting markedly improved condition; see Figs. 2 and 3.

**Table 6:** Treatment courses and prognosis of 18 highly suspected LAHLH patients receiving different initial treatment

No.	Age/sex	Diagnose	Initial treatment	Initial treatment response	Continuous treatment	Survival (days)	Prognosis
1	25/M	Highly suspected lymphoma	DA-EPOCH	CR	1 cycle EPOCH + splenic radiotherapy + 1 cycle MINE	190	Survival
2	43/F	Highly suspected lymphoma	DA-EPOCH	CR	3 cycles EPOCH + 2 cycles MINE + splenic radiotherapy	652	Survival
3	44/M	Highly suspected lymphoma	DA-EPOCH	CR	3 cycles DA-EPOCH + 3 cycles GDP + 1 cycle MINE	206	Died
4	71/F	Highly suspected lymphoma → T cell lymphoma	CHOP	CR	–	74	Died
5	55/M	Highly suspected lymphoma → T cell lymphoma	CHOP	CR	1 cycle CHOP + splenic resection + 1 cycle Hyper-CVAD-A + 2 cycles MINE	596	Died
6	32/M	Highly suspected lymphoma → T cell lymphoma	DA-EPOCH	CR	1 cycle DA-EPOCH + allo-HSCT	363	Died
7	41/F	Highly suspected lymphoma	EPOCH	CR	4 cycles EPOCH + 1 cycle L-GDP	437	Died
8	48/F	Highly suspected lymphoma	DA-EPOCH	NR	–	33	Died
9	60/M	Highly suspected lymphoma → B cell lymphoma	DA-EPOCH	CR	5 cycles DA-EPOCH + 2 cycles DHAP + 1 cycle MINE	264	Died
10	25/F	Highly suspected lymphoma → PTCL	EPOCH	CR	5 cycles L-GDP + 3 cycles-Hyper-CVAD-A +auto-HSCT	756	Survival
11	36/M	Highly suspected lymphoma	CHOP	NR	–	35	Died
12	65/F	Highly suspected lymphoma	CHOP	CR	2 cycles CHOP	115	Died
13	69/M	Highly suspected lymphoma	Hyper-CVAD-A	NR	–	92	Died
14	26/M	Highly suspected lymphoma	High-dose corticosteroid and IVIG	NR	–	12	Died
15	29/M	Highly suspected lymphoma	High-dose corticosteroid and IVIG	NR	–	27	Died
16	18/M	Highly suspected lymphoma	High-dose corticosteroid and IVIG	NR	–	8	Died
17	32/M	Highly suspected lymphoma	High-dose corticosteroid and IVIG	NR	–	17	Died
18	39/F	Highly suspected lymphoma	High-dose corticosteroid and IVIG	NR	–	15	Died

F, female; M, male; PTCL, peripheral T cell lymphoma; Hyper-CVAD-A, cyclophosphamide, mesna, vincristine, doxorubicin, dexamethasone; CHOP, cyclophosphamide, doxorubicin, vincristine, prednisolone; EPOCH, etoposide + CHOP; DA-EPOCH, dose-adjusted EPOCH; IVIG, intravenous immunoglobulin; GDP, gemcitabine, dexamethasone, cisplatin; MINE, mitoxantrone, ifosfamide, mesna, etoposide; l-GDP, l-asparaginase + GDP; CR, clinical response; NR, no response

**Fig. 4:**
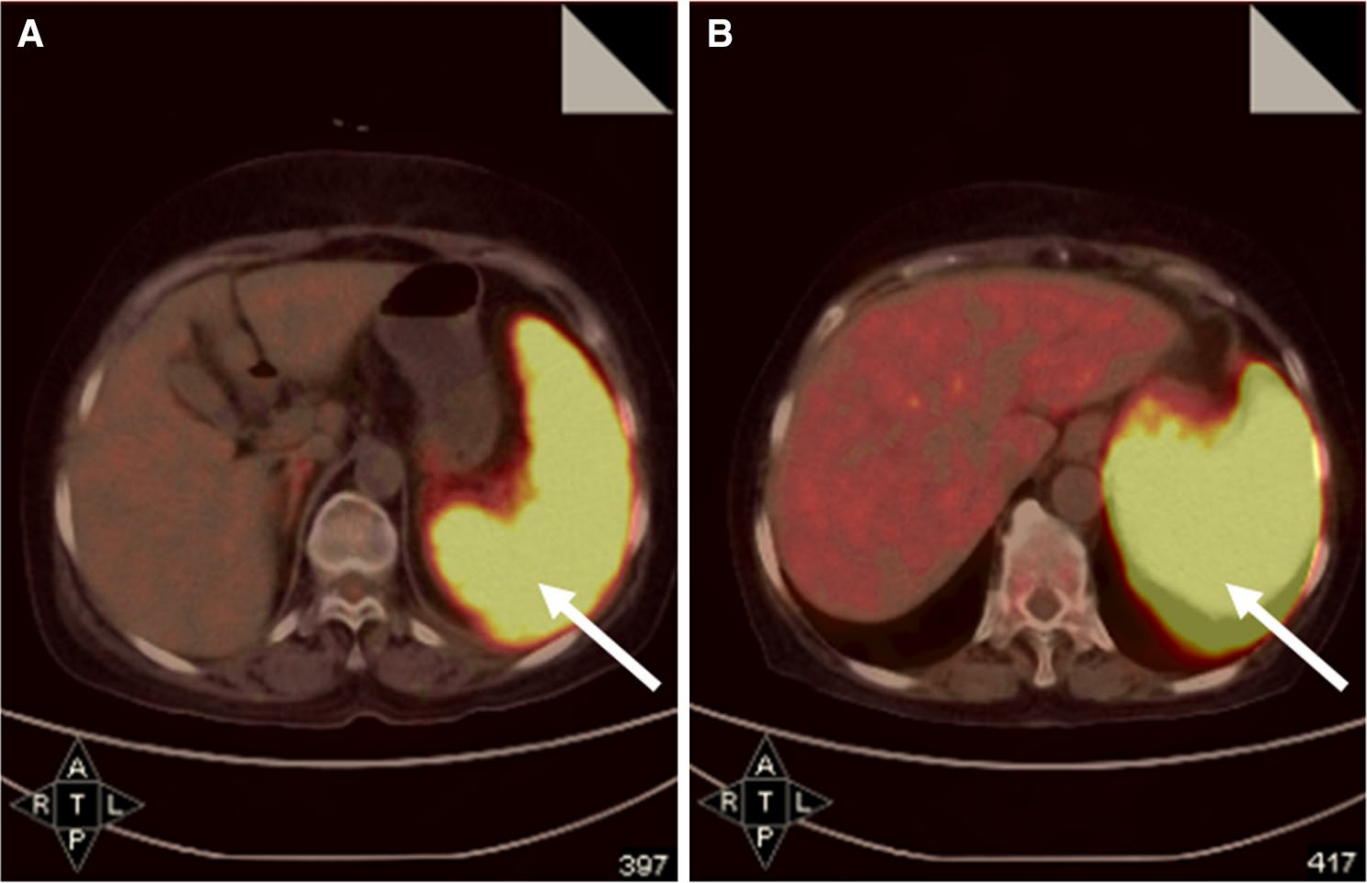
Comparison of PET–CT images of patient no. 15 before and after immunosuppressive therapy. **a** Initial PET–CT scan revealed FDG-avid splenomegaly with a SUV_Sp_ of 8.6 (*arrow*). **b** After receiving immunosuppressive therapy, post-therapeutic PET–CT revealed slightly smaller size in spleen; however, diffuse intense FDG uptake was higher than before with a SUV_Sp_ of 15.6 (*arrow*). In addition, new patchy FDG-avid lesions in humeri were shown, suggesting poor efficacy (not shown here). This patient survived for only 27 days

Five patients with highly suspected LAHLH were initially administered high-dose corticosteroid plus IVIG (immunosuppressive therapy). All of them showed no response and died within 4 weeks. One of them (patient no. 15) received post-treatment PET–CT after receiving high-dose corticosteroid plus IVIG. Although his spleen was slightly smaller than before which could be observed on PET–CT re-examination, diffuse intense FDG uptake was not relieved at all. In addition, diffuse patchy heterogeneous pattern of FDG uptake on his humeri was displayed, suggesting poor efficacy (shown in Fig. 4).

### Diagnostic performance of PET–CT for detection of lymphoma in HLH patients

Among 18 HLH patients who were highly suspected as lymphoma by PET–CT, five patients (patient no. 4, 5, 6, 9, and 10) ultimately received pathological confirmation of lymphoma. Of these, one had peripheral T cell lymphoma, one had B cell lymphoma, and three had T cell lymphoma (see Table 6). Lymphoma was confirmed by bone marrow biopsy in patient no. 4, 6, and 9 after initial chemotherapy; by bone marrow biopsy in patient no. 5 in the end stage of the disease; and by a second cervical lymph node biopsy in patient no. 10. Therefore, these cases confirmed that PET–CT plays an important role in detection of lymphoma in HLH patients without pathological basis. PET–CT and pathological examination are consistent in the diagnosis of lymphoma to a certain extent. Pathological evidence was unavailable for the other 13 patients. Among them, five out of eight patients who accepted lymphoma-chemotherapy showed satisfactory clinical response, including normal body temperature, reduction in spleen and lymph node size, recovery of blood cell counts, normal fibrinogen, decreasing levels of ferritin, LDH, transaminases, and bilirubin. None of the five patients on immunosuppressive therapy showed clinical response (see Table 6). Their better efficacies with lymphoma-chemotherapy than with immunosuppressive regimen indirectly showed the reliability of PET–CT to diagnose them as LAHLH. There is no doubt that pathological biopsy is most important in the management of adult HLH patients. But for those HLH patients who are in critical condition and are unavailable to pathological biopsy and other etiological factors, PET–CT might be of help in screening for potentially undetected lymphoma. Certainly, conclusions on primary triggers should be drawn carefully. Decisions about etiological factors and more appropriate therapies should be made by integrating pre-treatment PET–CT with clinical manifestations and laboratory results.

## Discussion

First described by Farquhar and Claireaux in 1952, HLH has a rapidly fatal clinical course. Familial HLH occurs due to known genetic defects (Ahn et al. 2010; Farquhar and Claireaux 1952), while secondary HLH may occur in association with severe infections, autoimmune diseases, or malignancies. However, the etiology distributions of secondary HLH vary a lot in different researches. A seminar paper summarized the causes of 2197 adult HLH, reporting about 50 % of infection and 57 % of neoplasms associated cases (Ramos-Casals et al. 2014). In a study of adult HLH patients, the causative factors were EBV (53.3 %), idiopathic (23.3 %), lymphoma (13.3 %), and other causes, with overall response rate of 50 % and mortality of 50 % (Ahn et al. 2010). In a recent study of 69 patients with secondary HLH from a single center of China, unexplained causes were responsible for 36.2 % of the cases, followed by infectious (33.3 %), lymphoma (21.7 %), and autoimmune disorders (7.2 %) (Li et al. 2014). In a previous study of our group, which introduced the application of COP regimen in treating sHLH, 46.7 % were infection-associated HLH, 26.7 % were cases with the absence of apparent underling diseases, 13.3 % were LAHLH, and 13.3 % were autoimmune disease-associated ones. In a recent retrospective study on sHLH from causes other than lymphoma, EBV infection (69.6 %) and idiopathic causes (26.1 %) consisted the main part of the whole (Park et al. 2012). As a consequence, we believe that lymphoma and unexplained ones account for a large part of sHLH. In our presented retrospective analysis, 14 patients (31.1 %) were classified as LAHLH, 5 (11.1 %) as EBV infection-associated HLH and 16 (35.6 %) as unexplained HLH. Since only newly diagnosed HLH patients who had undergone PET–CT before treatment were enrolled in our study, there may be statistic bias in the distribution of etiology in our paper. Although due to various etiologies, patient characteristics, severity of complications, or underlying medical conditions, the clinical responses and mortality rates may differ a lot among different studies, the overall prognosis of sHLH is quite poor.

Diagnostic criteria have been well proposed and updated; however, it is still challenging to make a diagnosis of HLH since most of the clinical and laboratory features are non-specific. Distinguishing extremely ill patients with multiorgan failure from HLH is difficult, since they have many common features (Tothova and Berliner 2014). Additionally, some patients could be diagnosed with sHLH in accordance with the diagnostic criteria, but no primary disease could be determined. Some other patients showed enlarged lymph nodes in the depth of the body or splenomegaly from imaging examinations. In the absence of enlarged superficial nodes, biopsies were unavailable. Biopsies of deep lymph nodes (such as para-aortic or retroperitoneal lymph nodes) or splenic resection were not recommended. Forced surgery could accelerate or even directly lead to death.

The imaging manifestations of HLH, which have lots of overlap in inflammation and hematological malignancy, are unspecific. Many researches have reported increased FDG uptake level in the spleen of patients with hematopoietic diseases and various infectious, like malignant lymphoma, acute virus infection, and infectious mononucleosis (Liu 2009; Lustberg et al. 2008; Sheehy and Israel 2007; Tomas et al. 2000). In a case report of T cell lymphoma-associated HLH with liver involvement, PET–CT showed diffuse intense FDG uptake in the enlarged liver and spleen, with systemic FDG-avid lymphadenopathy. In a study of 766 lymphoma patients, scholars indicated that with the exception of extranodal marginal zone lymphoma and small lymphocytic lymphoma, most lymphoma subtypes have high 18 F-FDG avidity (Weiler-Sagie et al. 2010). In our present study, the most common feathers of sHLH patients were splenomegaly and/or lymphadenopathy with various degrees of increased FDG uptake and bone lesions with increased FDG uptake. Inflammatory changes in the chest were generally observed as well, including pneumonia, atelectasis, pleura thickening, pleura adhesion, and hydrothorax. High levels of cytokines have been identified in the pleural fluid of patients with virus-associated HLH (Ohga et al. 1993). Therefore, we though that strong cytokine storm in lung tissue or infection due to neutropenia or dysfunction of immune system may result in these changes. Other common PET–CT feathers comprised inflammatory changes in the gallbladder, sinusitis, brain lesions, hepatomegaly, and lower density of the heart chamber than the ventricular wall which reflected an anemic state. Chung (2007) presented brain histology findings of HLH in the early stage, including lymphocytic leptomeningeal infiltration with hemophagocytosis and proliferation of histiocytes in the meninges, brain parenchyma, and perivascular spaces. They also observed calcification and necrosis of putamen, internal capsule, thalamus, and demyelination of white matter on MRI during disease progression. Fitzgerald and MacClain (2003) reported MRI manifestations of 18 HLH patients, mainly included abnormal signals of white matter, brain atrophy, and cerebral ventricle dilatation. In our study, a total of 13 cases had brain lesions, involving five cases reduced and one case increased uptake level of cerebral cortex, four cases infarction, two cases atrophy, and one case cerebral aneurysm. However, none of the six cases who had changed FDG uptake level in cerebral cortex showed clinical manifestations of central nervous system infiltration.

Further investigation in clinical or laboratory characteristics of sHLH may be of benefit to make early differential diagnosis. Our previous study found that SUV_max_ may especially play impotent role in differential diagnosis of sHLH (Zhang et al. 2012). In the present study, we conclude that not only SUV_max_, but also SUV_Sp_, SUV_BM_, SUV_LN_, SUV_LN/Li_, and SUV_max/Li_ may be of significance in the differential diagnosis of LAHLH. We comparatively analyzed the PET–CT features of LAHLH and non-LAHLH and found that patients in LAHLH group had a markedly higher level of SUV_Sp_, SUV_BM_, SUV_LN_, SUV_max_, SUV_LN/Li_, and SUV_max/Li_ than those in non-LAHLH group (*p* = 0.003, *p* = 0.034, *p* = 0.003, *p* < 0.001, *p* = 0.039, and *p* = 0.035, respectively). As a result, SUV_max_ higher than 5.5, SUV_LN_ higher than 3.3, and SUV_Sp_ higher than 4.8 could be used as differential diagnosis to distinguish LAHLH from sHLH, wherein SUV_max_ had a high diagnostic accuracy (AUC = 0.933), and the other two parameters had moderate diagnostic accuracy (AUC = 0.834, AUC = 0.832, respectively).

Other laboratory characteristics that are benefit to the differential diagnosis of LAHLH were reported. Li et al. (2014) had analyzed 69 adult HLH patients and found that the percentages of patients with Fbg < 1.5 g/L, PLT < 40 × 10^9^/L and LDH > 1000 U/L in the LAHLH group were significantly higher than those in non-LAHLH patients. However, our result showed no significant differences in Fbg, PLT, and LDH level between LAHLH and non-LAHLH group (data not shown). In addition, we found that the percentages of patients who had multiple lymphadenopathy or multiple bone lesions in LAHLH group were significantly higher than those in non-LAHLH group (*p* = 0.004, *p* = 0.016, respectively). Solely enlarged lymph node with increased FDG uptake occurs in patients with reactive lymphoid hyperplasia or lymphoid tuberculosis. Two different situations of bone lesions were observed, including diffusely increased FDG uptake in systemic bones and increased FDG uptake in focal bone regions. Diffuse increased SUV uptake level in bones especially cancellous bones may reflect increased reactivity of hematopoiesis. According to a result of Inoue et al. (2009), BM F-18 FDG uptake depended on patient age and inflammatory activity, indicating BM activation. Some scholars maintained that the persistent secretion of high cytokine might lead to clinical signs and symptoms as well as increased F-18 FDG uptake in the BM and spleen (Inoue et al. 2009; Liu 2009). In our study, the incidence of multiple lymphadenopathy (accompanied by increased FDG uptake) and multiple bone lesions in LAHLH patients were significantly higher than those in non-LAHLH group. Therefore, we believe that multiple sites of lymphadenopathy and bone lesions with increased FDG uptake may be prompted to multiple invasion and metastasis of lymphoma, thus indicating lymphoma-related HLH than simply increased SUV_BM_ and SUV_LN_.

F18-FDG PET–CT can not only help to the differential diagnosis of sHLH, but also help to the assessment of therapeutic response (Cronin et al. 2010). Convincing evidence showed that persistent FDG uptake after 2–4 cycles of chemotherapy is associated with poor outcome in Hodgkin lymphoma and non-Hodgkin lymphoma (Hutchings et al. 2006; Mikhaeel et al. 2005). And Suga et al. (2010) reported, in a case of T cell lymphoma-associated HLH, sites of hypermetabolic abnormalities which existed at first visit and disappeared after taking 6 months of chemoimmunotherapy indicating remission of disease. There were five highly suspected LAHLH patients in our study received a second PET–CT scan after lymphoma-chemotherapy, of whom the PET–CT parameters had markedly decreased.

Since HLH can be rapidly fatal without specific intervention, it is recommended that treatment be started when there is a high clinical suspicion, even when results of some diagnostic studies are still pending (Filipovich 2009). Park et al. (2012) analyzed the treatment outcome and prognostic factors of sHLH and found that patients whose treatment began within 5.5 days of the first hospital visit were more likely to survive. Therefore, earlier therapeutic intervention is the key to save lives. For unexplained sHLH patients who account for a relatively large proportion, to start therapy after blindly pursuing pathological evidences may miss the best timing of treatment.

Various treatment strategies have been reported, such as HLH-94 protocol, HLH-2004 protocol, CHOP regimen, steroid pulse therapy, IVIG, TNF-α monoclonal antibody (Henzan et al. 2006), CD25 monoclonal antibody (Olin et al. 2008), and hematopoietic stem cell transplantation. However, due to the complexity of sHLH itself, treatment outcomes for adult cases have seldom been reported and varied widely. Since early application of etoposide with sufficient dose has a unique effect on EBV-associated HLH, etoposide-containing immunochemotherapy, like HLH-2004, has become a recommended treatment for childhood EBV-associated HLH (Imashuku et al. 1999, 2001). After early use of HLH-2004 protocol, hypercytokinemia was quickly under control, and clonal proliferative T/NK cell and activated macrophages were removed. With IVIG, infection-associated HLH patients seemed to benefit most with response rate of 78 %, but lymphoma-associated patients seemed to be largely ineffective nevertheless (Larroche et al. 2000). It was proposed in a review that corticosteroids alone or combined with cyclosporin A and/or intravenous immunoglobulin might be appropriate for low-risk situations, like RAHLH; early etoposide, antithymocyte globulin, and polychemotherapy were recommended for high-risk or refractory/relapse cases, such as Hodgkin’s disease or non-Hodgkin’s lymphoma (NHL) (Emmenegger et al. 2005). Although chemo-approach and etoposide were suggested, the five patients who accepted immunosuppressive therapy were unwilling to accept chemo-drugs due to lack of pathological evidences to lymphoma. Presented hyperbilirubinemia and dysfunction of liver in these patients, and high risk of infection, disease reactivation, and secondary malignancies associated with etoposide use (Ramos-Casals et al. 2014), made the patients object to etoposide. Till now, standardized and conformably approved regimens for adult HLH have not been well established.

Considering that CHOP regimen is a standard chemotherapy for aggressive NHL, it is considered as an effective regimen to treat HLH combined with malignant lymphoma (Shin et al. 2008). Although based on a small number of patients in the current study, lymphoma-chemotherapy shows a therapeutic potential in highly suspected LAHLH patients. In our study, compared with immunosuppressive therapy, highly suspected LAHLH patients who initially received lymphoma-chemotherapy have significantly higher CR rate and relatively favorable prognosis. Although it is recommended that the first goal of therapy in adult HLH patients is to suppress the unregulated hyper-inflammation, while the second is to identify and treat the underlying triggers (Kleynberg and Schiller 2012). However, our results indicate that lymphoma-chemotherapies that treat the underlying lymphoma should be the first intervention for highly suspected LAHLH. Immunosuppressive or cytokine-regulating therapies could not reduce the soaring cytokines from the root causes.

In conclusion, we showed that PET–CT plays an important role in confirming the diagnosis of LAHLH without pathologic evidence. Patient in LAHLH group had remarkably higher levels of SUV_Sp_, SUV_BM_, SUV_LD_, SUV_max_, SUV_LD/Li_, and SUV_max/Li_ than those in non-LAHLH group. In addition, sHLH patients who presented in PET–CT images with multiple lymphadenopathy and/or multiple bone lesions accompanied by increased FDG uptake were more likely to be LAHLH. For the initial treatment of these highly suspected LAHLH, lymphoma-chemotherapy, rather than immunosuppressive therapy (high-dose corticosteroid plus IVIG), may have a relatively favorable effect and better clinical outcomes. In conclusion, for sHLH patients without etiological evidence, PET–CT has high diagnostic value in detection of lymphoma. Once highly suspected as LAHLH by PET–CT, lymphoma-chemotherapy should be initially applied to improve their prognosis.
